# The growth of *Scenedesmus sp.* attachment on different materials surface

**DOI:** 10.1186/s12934-014-0142-z

**Published:** 2014-10-01

**Authors:** Xiaolin Chen, Tianzhong Liu, Qiang Wang

**Affiliations:** Insitute of Oceanology, Chinese Academy of Sciences, Qingdao, 266071 China; Key Laboratory of Biofuel, Qingdao Institute of Bioenergy and Bioprocess Technology, Chinese Academy of Sciences, Qingdao, 266101 China; College of Chemistry and Pharmaceutical Sciences, Qingdao Agricultural University, Qingdao, 266109 China

**Keywords:** *Scenedesmus sp.*, Attachment, Supporting surface, Biomass productivity, Growth

## Abstract

**Background:**

Microalgae has been concerned as a potential source of biodiesel in the recent years. However, it is costly to harvest microalgae as it is commonly cultured in water and the cells are too small to harvest. In order to reduce the cost of cultivation and harvesting, it is important to improve the biomass productivity of microalgae. Here, we utilized the attachment method to culture microalgae to cut off the cost of culture and harvest.

**Results:**

In this paper, various supporting surface with different hydrophility including polyvinylidene fluoride (PVDF), polyacrylonitrile (PAN), polysulfone (PS), which are not easy to be degraded in the culture medium, were used for microalgae culture by the attachment method. The results showed that PVDF supporting cloth was suitable for the algae growth, and its average biomass productivity was to 4.0 g/m^2^/day. Furthermore, a series of PVDF concentrations were tested, and cloth treated with 3% or 5% PVDF solution was better for the algae culture. In addition, Polyvinylpyrrolidone (PVP) with different molecular weight was added to the PVDF solution as porogens to produce rough surface. And the addition of PVP resulted in better growth with 6.0 g/m^2^/day of average biomass productivity.

**Conclusion:**

This attachment method makes the harvest of microalgae easy and energy-saving, because the microalgae grow on the supporting material and is easy to be scraped. The results indicate that the PVDF-treated cloth is a potential alternative for the microalgae attachment culture.

## Background

The world has been confronted with an energy crisis in recent decades, associated with irreversible depletion of traditional fossil fuels. Their use as major form of energy is indeed unsustainable, further to accumulation of greenhouse gases in the atmosphere that brings about global warming [1]. Compared to conventional diesel, biodiesel generally contains a higher level of oxygen and lower levels of sulfur and nitrogen and therefore, less SOx, NOx, CO, benzene and toluene are released upon combustion. [2,3]. Currently, the most widely available form of biodiesel comes from oil crops such as palm, oilseed rape, soybean and microalgae. For all the biodiesel sources, microalgae has attracted interest as a source of high-lipid materials to produce biofuel, since photosynthetic conversion is an efficient and alternative process and avoids any competition with food crops [4].

However, to date, the commercial viability of producing biodiesel from microalgae is still marginal due to high production cost. This mainly includes cultivation cost, harvesting cost and lipids extraction cost. In order to cut off the cost of biodiesel production, the above three cost must be saved. Firstly, to reduce the cultivation and harvesting cost, it is important to improve the biomass productivity of microalgae.

Microalgae cultivation is typically performed in open ponds or enclosed photo bioreactors in which algal cells are grown in suspension. The biomass concentration in the culture solution is common in the range of 0.1-1 g/L [5–7]. This directly causes high harvesting cost. In addition to the above culturing methods, an attachment algal culture system such as an algal turf scrubber (ATS) is another culture configuration in which benthic algae grow on the surface of solid supporter. This culture system has been successfully used for growing filamentous microalgae for removing nutrients from animal wastewater and growing *Chlorella sp.* on different materials [8–10,7]. However, the productivity of *Chlorella sp.* attachment on supporting surface of polystyrene foam was very low [7]. If this was as the biodiesel feedstock, the productivity should be further improved. Therefore, in this paper, we adopted the attachment algal growth concept to grow *Scenedesmus sp.* on different supporting surface with the purpose of finding good material to grow microalgae and improve the productivity by attachment culture.

## Results and discussion

### The growth of *Scenedesmus sp.* on different supporting materials

**Figure 1:**
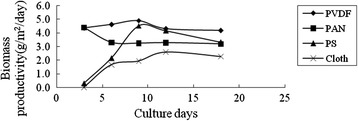
**The effect of different supporting materials on the growth of** ***Scenedesmus sp..***

**Figure 2:**
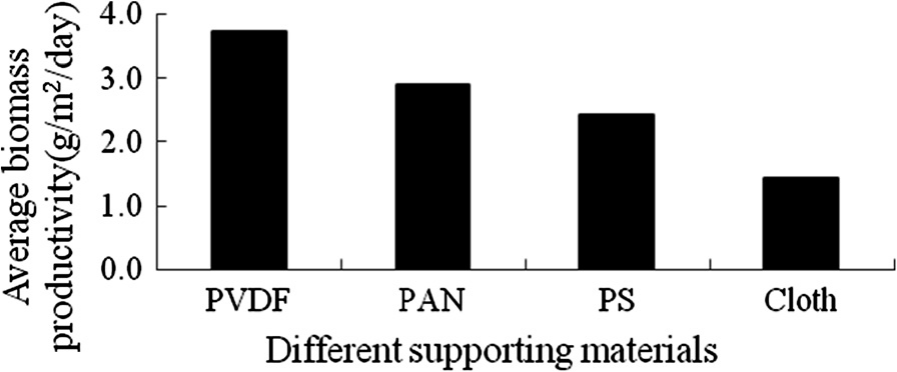
**The average dried weight of the** ***Scenedesmus sp.*** **of different supporting materials.**

**Figure 3:**
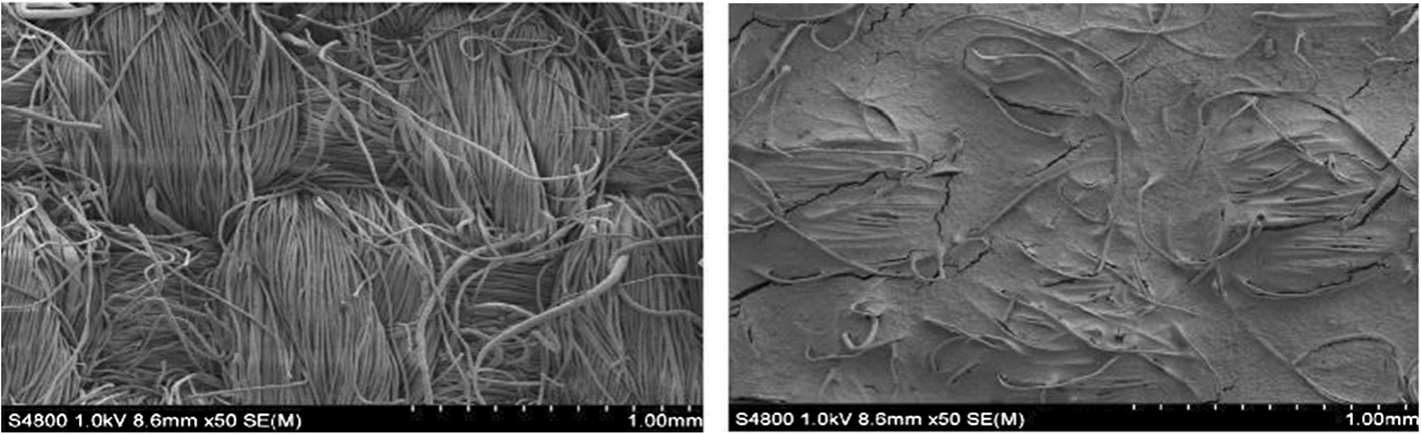
**The SEM of the surface of the cloth and PVDF supporting surface.**

The algae attachment experiments were conducted by incubating algal cells in growth chambers with different supporting materials as described in “Methods”. Figure 1 and Figure 2 showed the growth of incubated algal cells on different supporting materials. During the whole culture process, the biomass productivity of the algal cells on PVDF, PAN and PS was better than that of the untreated cotton cloth, probably because PVDF, PAN and PS filled the large pore on the cloth which made the cells grow on the surface instead of growing into the cloth which is difficult to harvest (Figure 3 ). In addition, the biomass productivities of PVDF and PAN were relatively stable. While that of the PS and cloth was unstable, it took about 10 days to obtain the highest biomass productivity. After 10 days, the biomass productivity decreased. From the results of the average biomass productivity, the algal cells on the PVDF supporting surface showed the best growth (3.74 g/m^2^/day). Here, the biomass productivity is a measurement of net biomass productivity ( the difference value between the whole harvested biomass and the inoculums).

**Figure 4:**
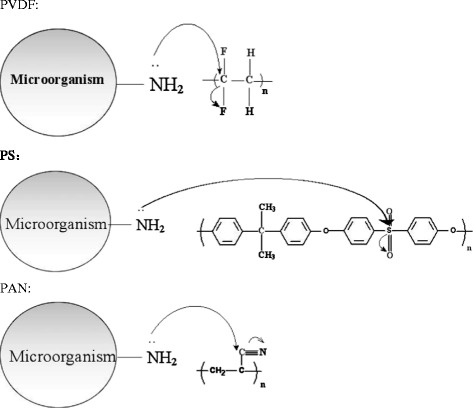
**Attachment on the surface of different supporting materials.**

The above results may be explained by the chemical structure of the supporting materials and the degree of swelling (Sw). As shown in Figure 4, some of the active microalgae protein in the outer of the cell wall, for example alkylation of the amino group supplied free electrons to the supporting materials [11–13]. Therefore, the ability of accepting electrons directly determined the attachment of microalgae onto the supporting surface. The electropositive of the carbon atom in C-F of PVDF is stronger than that of sulfur atom in S = O of PS and that of carbon atom in C ≡ N of PAN, therefore, for the ability of accepting electrons, the former is stronger than the latter.

Sw of the supporting materials were shown in Table 1. The Sw of different supporting materials might indicate which materials could supply adequate nutrition to microalgae. The bigger Sw, the more obtained nutrition. From the results of Table 1, the Sw of the cotton cloth is the maximum, PVDF followed. And the Sw of PS and PAN were less. Although the Sw of the cloth is the biggest, the biomass productivity was the lowest. Probably, the pores on the surface of the cloth was much bigger than the microalge cell, so there were many cells growing into the pores of the cloth, and easy to be taken away by the flowing culture medium. The pores on the surface of the treated cloth by PVDF, PS and PAN were smaller than that of the cloth. Consequently, there were relatively less microalgae growing into the pores. Generally speaking, the biomass productivity of the cell is closely related to characteristics of the supporting materials. For the above materials, PVDF was more suitable as supporting material for the following experiments.

**Table 1:** **The Sw of different supporting materials**

	**Cloth**	**PVDF**	**PS**	**PAN**
Sw	150.50%	92.78%	85.43%	72.85%

### The effects of different concentrations of PVDF solution on the growth of *Scenedesmus sp.*

**Figure 5:**
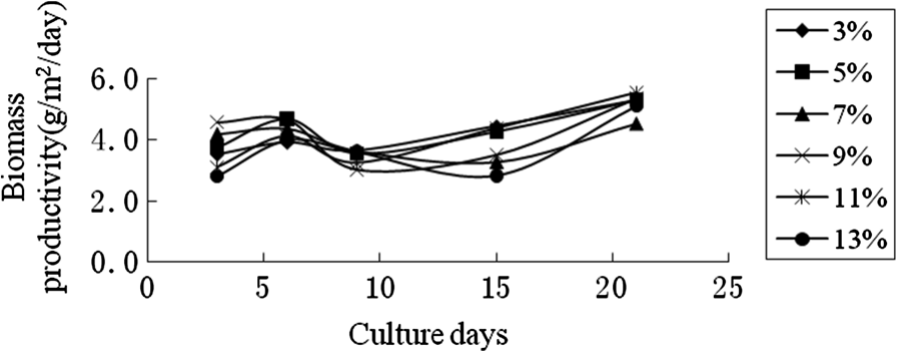
**The effect of different amounts of PVDF on the growth of** ***Scenedesmus sp..***

**Figure 6:**
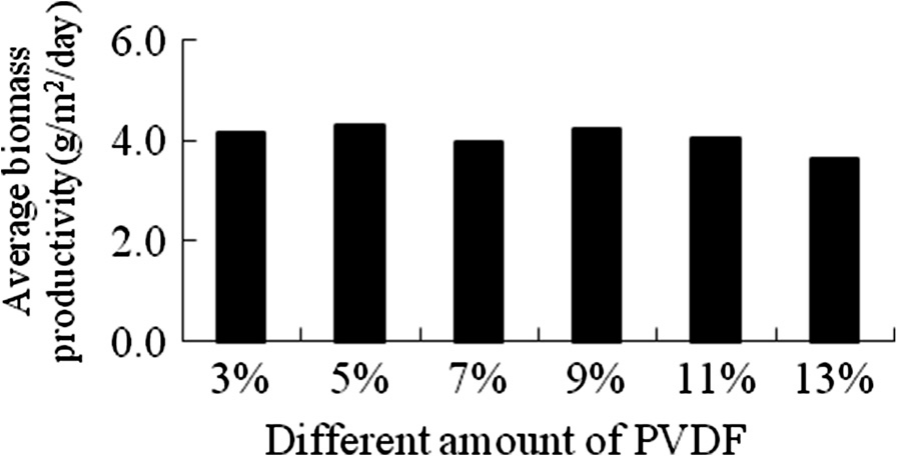
**The average dried weight of the** ***Scenedesmus sp.*** **on different amount of PVDF.**

Figures 5 and Figure 6 showed the biomass productivity and average biomass productivity of *Scenedesmus sp.* on cloth treated with different concentration of PVDF solution. The results indicated that it was obviously different for the biomass productivity among different concentration of PVDF solution, which is possible due to the pores size on the surface of cloth. 3% or 5% PVDF can satisfy the needs for the microalgae growth from the results. The average biomass productivity for the 13% PVDF group was the lowest, because the treated cloth became stiff which possibly affected the water and nutrients retention. If this material is applied to the large-scale algae cultivation, in order to save the cost, 3% PVDF can be used to form the supporting material.

### The effects of addition of PVP porogens with different molecular weight on the growth of *Scenedesmus sp.*

**Figure 7:**
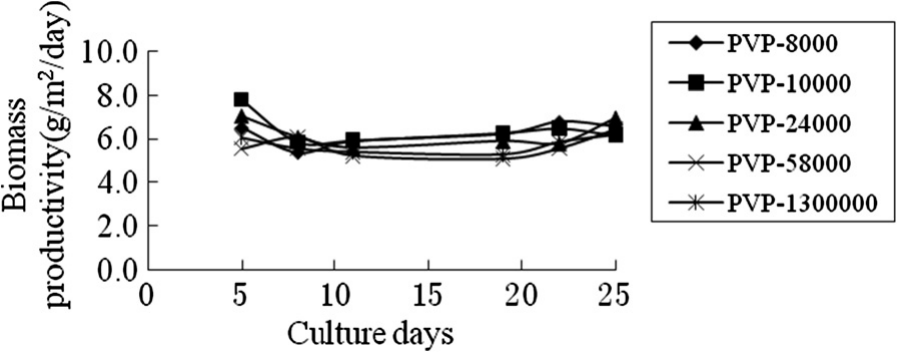
**The effect of different molecule PVP on the growth of** ***Scenedesmus sp..***

**Figure 8:**
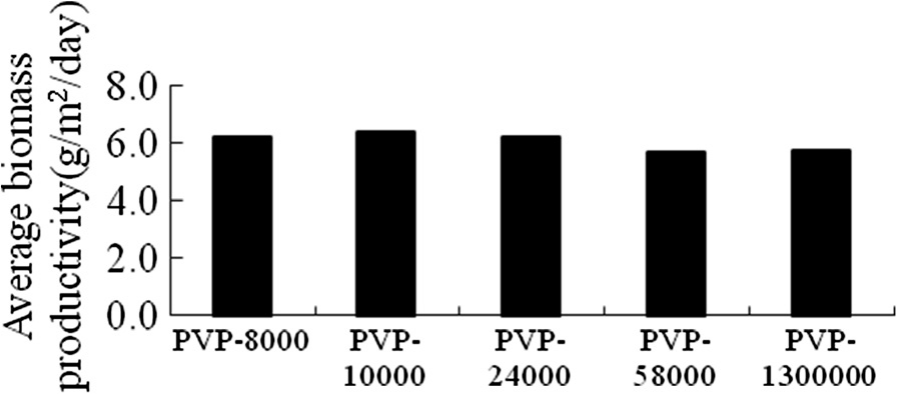
**The average dried weight of** ***Scenedesmus sp.*** **for different molecule PVP.**

In theory, if the pores on the supporting surface satisfy the growth of algae, the rougher surface will led to the more favorability of microalgae attachment and growth. In order to prove this opinion, different molecular weight porogens of Polyvinylpyrrolidone (PVP) were added to the PVDF solution. And the biomass productivity and average biomass productivity of *Scenedesmus sp.* were shown in Figure 7 and Figure 8. For the whole culture process there was obviously different among all the molecular weight porogens, and the algae growth of the groups PVP-8000, PVP-10000, PVP-24000, was slightly better than that of higher molecular PVP. This was possibly because the formed pores were closer to the size of *Scenedesmus sp.* Cell [14]. As expected, the addition of porogens resulted in better growth of algae (about 6.0 g/m^2^/day of average biomass productivity) than that of non-addition (about 4.0 g/m^2^/day of average biomass productivity).

This paper introduced attachment porous supporting material to cultivate *Scenedesmus sp.*. And the results showed that PVDF-PVP-8000 was good for the growth of *Scenedesmus sp.*. In addition, in our lab, other algae such as *Spirulina platensis* and *Tribonema sp.* have been successfully cultivated (not published). However, it need fatherly study whether this supporting material or the treatment method was fit for other kinds of microalgae such as diatoms and dinoflagellates that are sensitive to shear stresses. In spite of this, it is important step for the cultivation of microalgae, because most of algae was fixed on the supporting material and only medium was circulated, which was beneficial for the incubation and harvest of microalgae. Therefore, it was suitable for the commercialization because the incubation and harvest was energy-saved. In addition, the culture system was different from open ponds and enclosed photobioreactors including thin-layer photobioreactor which has been used more than 20 years used for commercial production of algal biomass in The Institute of Microbiology, Czech Republic [15–18]. This system avoided high energy consumption of separation cells from culture mixture because most of the cells grew on the supporting material and the harvesting step only need scrapping tool, but the microalgae cultured in the other photobioreactors still need centrifugation, ultrafiltration or other harvesting methods to separate cells from the suspension mixture.

## Conclusions

In this paper, we adopted the attachment algal growth concept to grow *Scenedesmus sp.* on different supporting surface with the purpose of finding good material to grow microalgae and improving the productivity. The results showed that cloth treated with 3% or 5% PVDF solution was appropriate material for the algae growth. And the porogen of PVP with lower molecular weight added to the PVDF solution resulted in better growth than that of non-addition group (about 6.0 g/m^2^/day of average biomass productivity via about 4.0 g/m^2^/day of average biomass productivity). The biomass productivity is higher than the reported attachment culture (0.26 g/m^2^/day) [7], which indicated that PVDF treated cloth is an good attachment material for microorganism culture because of its good attachment and desirable physical properties.

## Methods

### Algae strain and subculture

The used alga was *Scenedesmus sp.* donated from Arizona State University. This green microalgae was chosen as a test organism because it is fast growing, easy culture and high oil production [19–22,5]. Algae cells were maintained in BG11 culture medium(NaNO_3_ 10 ml/L stock solution 15.0 g/L dH_2_O; K_2_HPO_4_ 10 ml/L stock solution 10.0 g/L dH_2_O; MgSO_4_.7H_2_O 10 ml/L stock solution 7.5 g/L dH_2_O; CaCl_2_.2H_2_O 10 ml/L stock solution 3.6 g/L dH_2_O; Citric acid 10 ml/L stock solution 0.6 g/L dH_2_O; Ferric ammonium citrate 10 ml/L stock solution 0.6 g/L dH_2_O; EDTANa_2_ 10 ml/L stock solution 0.1 g/L dH_2_O; Na_2_CO_3_ 10 ml/L stock solution 2.0 g/L dH_2_O; H_3_BO_3_ 1 ml/L stock solution 2.86 g/L; MnCl_2_.4H_2_O 1 ml/L stock solution 1.86 g/L; ZnSO_4_.7H_2_O 1 ml/L stock solution 0.22 g/L; Na_2_MoO_4_.2H_2_O 1 ml/L stock solution 0.39 g/L; CuSO_4_.5H_2_O 1 ml/L stock solution 0.08 g/L; Co(NO_3_)_2_.6H_2_O 1 ml/L stock solution 0.05 g/L) before inoculums on the supporting surface.

### Attached algae culture

**Figure 9:**
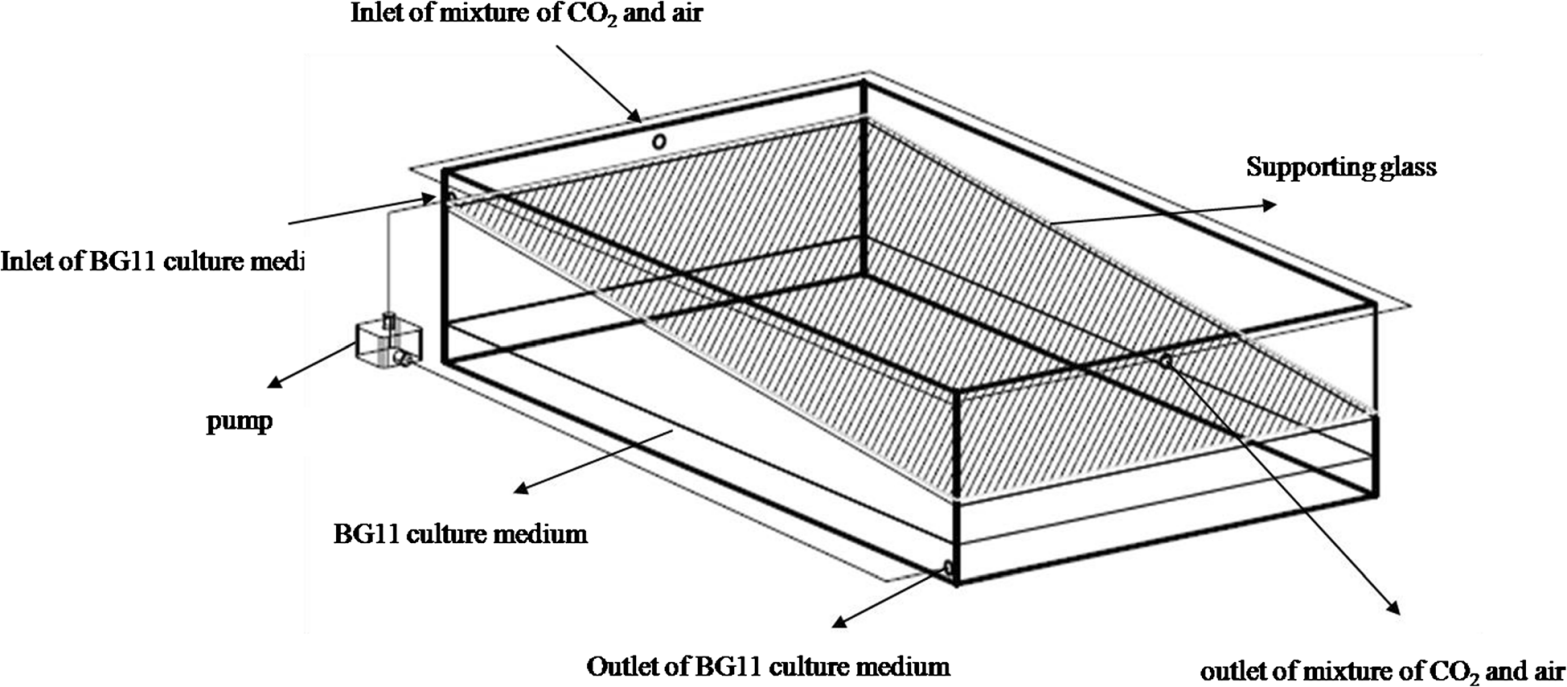
**Schematic of attachment algal culture system.**

Figure 9 showed the schematic of the attachment algae culture system. The supporting material was cut into 5 cm × 5 cm piece and fixed on the surface of the declining glass in the growth chamber. The supporting material was incubated by putting it in concentrated algae cell suspension (about 50 g/L) for 5 minutes. Then, the incubated supporting material was placed on the wet filter paper which was immobilized on the declining glass. And 1.8 L BG11 culture medium was put into the growth chamber. The culture medium was pumped from the bottom to the upper of the declining glass in order to keep the supporting materials moisture. And the mixture CO_2_ and air was aerated from the inlet to outlet. The growth chamber was continuously illuminated with cool white lights at about 50 μmol.S^−1^.m^−2^. It was placed at 20° from the horizontal plane. And the culture temperature was 25°C. Three replicates were carried out for every supporting material. And samples were taken every 3 days.

When the attached algae cells formed a thick “mat” on the surface, it was harvested by a glass blade. After harvesting, the substrate was placed into the growth chamber and the medium in the chamber was replaced with fresh BG11 culture medium. The remaining algal colonies on the surface can serve as inoculums for the next cycle of growth [8].

### Growth analysis

The biomass productivity were measured with gravimetric method. The harvested cells was filtered to pre-weighted 0.45 μm GF/C filter membrane(Whatman, England; DW_1_). The membrane with the cells was oven dried overnight at 105°C, cooled down to room temperature in a desiccators, and then to measure dry weight again (DW_2_). The biomass productivity (P, g m^−2^ d^−2^) was calculated as:

$$ \mathrm{P}=\left({\mathrm{DW}}_2\hbox{-} {\mathrm{DW}}_1\right)/0.0025\mathrm{t} $$

In which the 0.0025 represents the area of the supporting materials. “t” represents cultivated time (d).

### The preparation of different supporting materials

Different materials were tested as supporting materials for algae attachment, including polyvinylidene fluoride(PVDF), polyacrylonitrile (PAN), polysulfone (PS), which were not easy to degrade in the culture medium, and they have different hydrophilicity.

For this experiment, the casting solution was prepared by dissolving the above materials and polyvinylpyrrolidone (PVP) in N, N-dimethylformamide (DMF). The solution was over-coated on the surface of dried cotton cloth with a roller, and vaporized for 10 minutes at room temperature. Then, the composite materials were placed into de-ionized water for 30 minutes in order to get rid of water-soluble components. Finally, they were dried at 50°C for 24 hours.

### Characterization

The degree of swelling (Sw) of the above treated supporting material was calculated as follows:

1 $$ \mathrm{S}\mathrm{w}=\frac{{\mathrm{W}\hbox{-} \mathrm{W}}_0}{{\mathrm{W}}_0}\times 100\% $$

Where W and W_0_ denote the weights of the treated supporting material with absorbed water and the dry one, respectively. The time duration for swelling was 24 hours.

Scanning electron microscopy (SEM) was used to study the morphology of the surface of the cotton cloth and the surface of treated cotton cloth in order to evaluate the supporting materials. In the measurement, the supporting material was fractured in liquid nitrogen and then the fractured part was coated with a conductive layer of a sputtered gold. The surface of the supporting material was investigated using HITACHI S-4800.

### Statistical analysis

Statistical analysis was carried out using SPSS 11.0 software (SPSS Inc, Chicago, USA). ANOVA was performed to evaluate significance of individual differences with a probability threshold of 0.05, followed by a Post-Hoc Tukey test.
